# Simple methods for quantifying super-resolved cortical actin

**DOI:** 10.1038/s41598-022-06702-w

**Published:** 2022-02-17

**Authors:** Evelyn Garlick, Emma L. Faulkner, Stephen J. Briddon, Steven G. Thomas

**Affiliations:** 1 Institute of Cardiovascular Sciences, College of Medical and Dental Sciences, University of Birmingham, Edgbaston, Birmingham, B15 2TT UK; 2 Centre of Membrane Proteins and Receptors (COMPARE), University of Birmingham and University of Nottingham, Birmingham, UK; 3 Division of Physiology, Pharmacology and Neuroscience, School of Life Sciences, University of Nottingham, Nottingham, UK

**Keywords:** Super-resolution microscopy, Actin

## Abstract

Cortical actin plays a key role in cell movement and division, but has also been implicated in the organisation of cell surface receptors such as G protein-coupled receptors. The actin mesh proximal to the inner membrane forms small fenced regions, or ‘corrals’, in which receptors can be constrained. Quantification of the actin mesh at the nanoscale has largely been attempted in single molecule datasets and electron micrographs. This work describes the development and validation of workflows for analysis of super resolved fixed cortical actin images obtained by Super Resolved Radial Fluctuations (SRRF), Structured Illumination Microscopy (3D-SIM) and Expansion Microscopy (ExM). SRRF analysis was used to show a significant increase in corral area when treating cells with the actin disrupting agent cytochalasin D (increase of 0.31 µm^2^ ± 0.04 SEM), and ExM analysis allowed for the quantitation of actin filament densities. Thus, this work allows complex actin networks to be quantified from super-resolved images and is amenable to both fixed and live cell imaging.

## Introduction

Cortical actin is a heterogeneous distribution of dense actin filaments in close proximity to the plasma membrane which undergoes constant remodelling^1^ and contributes to cellular structure, migration, and division. Super resolution microscopy studies have indicated that cortical actin can lie between < 10 nm and a maximum of 20 nm from the membrane^2^. This close association between actin and the plasma membrane are in agreement with the picket fence model proposed by Fujiwara et al.^3^. This model suggests that actin forms small fenced regions, or ‘corrals’, within which membrane proteins can become transiently trapped (Supp. Figure 1). The cytoskeleton is decorated by transmembrane proteins, or ‘pickets’, which have been shown to directly slow local diffusion in the membrane, either by increased packing of lipids around the protein^4^, physical steric hindrance^5^ or through increased hydrodynamic interactions^6^. There is significant evidence that actin can regulate organisation of certain receptors within the membrane (e.g. serotonin 1a receptor^7^, α2A-adrenergic receptor^8^, and the LYVE-1 receptor^9^) but the precise mechanism by which this occurs has yet to be fully visualised. It would therefore be beneficial to image and analyse these actin corrals at high resolution.

Prior to the explosion of super resolution light microscopy techniques, electron microscopy was the best option available to image intricate actin networks at a high resolution. Fujiwara et al.^10^ showed corrals with a median length of 40 nm in PtK2 cells, but 230 nm in NRK cells, suggesting cell specific confinement areas. Electron microscopy however requires complex preparation which can induce artefacts, as well as having limited options for labelling molecules of interest. Single molecule localisation microscopy (SMLM) techniques can now resolve structures down to ~ 20 nm, almost rivalling EM, and therefore capable of resolving individual actin filaments in the dense mesh of the cortical actin^11^. For example, Photoactivated Localisation microscopy (PALM) imaging of mEOS-Lifeact was used by Sungkaworn et al.^8^ to demonstrate the corralling influence of actin on α2A-adrenergic receptors. Thus far, analysis of super resolved actin filaments largely focuses on single molecule techniques. Peters et al.^12^ developed a method of filament tracing dependent on localisation density, allowing analysis of filament length and branching. Pore analysis, focusing on spaces between filaments after image processing to enhance filamentous structures, has also been developed for SMLM images^13^. Machine learning techniques for point cloud data are also being developed to investigate filamentous structures and their relation to clustered points^14^, useful for investigating actin and receptor cluster relationships. Stimulated Emission Depletion (STED) microscopy and Structured Illumination Microscopy (SIM) techniques have a lesser resolving power than SMLM, but still provide at minimum a doubling of standard widefield resolution, and are therefore powerful tools for actin imaging studies. As a recent example, Stanly et al.^9^ used STED microscopy to image actin corrals in conjunction with the LYVE-1 receptor, reporting corrals with an average size of 100 nm–1.5 µm, each containing heterogeneously distributed receptor clusters.

As yet, the study of the structure and function of cortical actin has largely focussed on mechanical properties (as reviewed by Svitkina^15^). While super resolution light microscopy has been used to begin quantifying corral structure, application of ever evolving super resolution imaging strategies will provide further key insight. Live actin imaging especially has often relied on widefield and TIRF imaging, which are subject to the resolution limit of light microscopy (~ 200 nm) and therefore unable to resolve finer actin structures. Investigations have therefore largely been on a macro or meso scale, looking at cortical actin intensity, actin flow, or focussing on the large bundled stress fibres. It should not be assumed that all actin can or does contribute in a similar manner to membrane organization—stress fibres often sit a small distance away from the membrane and have very different functions to less bundled cortical actin. However, this does not preclude contribution of larger bundled filaments to membrane protein dynamics^16^.

Analysis of actin networks at a high resolution is a developing field. By focussing on the empty space the actin structure creates in SRRF and SIM images, as opposed to the filaments themselves, the work described here sets up analysis workflows that can be used to simply assess the cortical actin mesh in fixed cell super resolved images, as well as quantifying the response of the actin network to disruption.

## Results

### Generation of super-resolved images of cortical actin networks

SRRF, or Super Resolved Radial Fluctuations^17^, is a technique that can be used to generate super resolved images from multiple standard widefield, TIRF, or confocal frames. SRRF images of phalloidin stained actin networks in fixed A549 cells were obtained as described in the methods and reconstruction assessed for accuracy (Supp. Fig. S2). Fourier Ring Correlation (FRC) measurements show a significant increase in resolution over standard TIRF images, as previously reported^17,18^. Use of NanoJ-SQUIRREL^19^ to assess SRRF images indicated robust and accurate reconstruction, routinely reporting Resolution Scaled Pearsons (RSP) correlation coefficient values of > 0.95, indicating good reconstruction of SRRF images. Visualisation of error as a heat map indicated issues largely around the thicker filaments.

### Quantification of cortical actin networks

**Figure 1:**
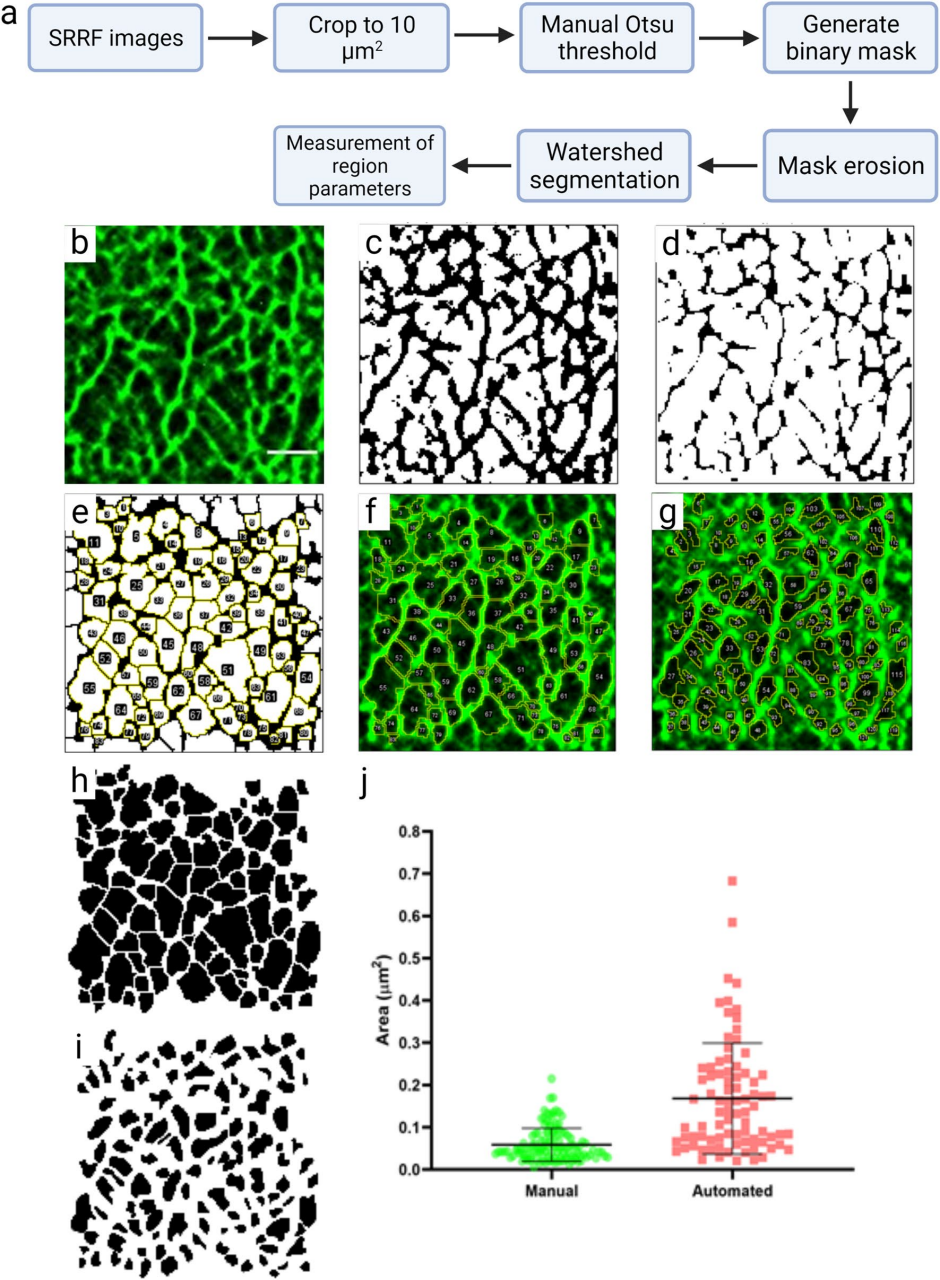
Development of corral analysis workflow. (**a**) Image analysis flow chart, detailing each step taken as illustrated in the following images. (**b**) Cropped SRRF image generated from 100 frames of TIRF data. (**c**) Manually selected Otsu threshold to exclude background pixels. (**d**) Erosion applied to (**c**). (**e**) Watershed segmentation and corral identification (yellow indicates borders of each individually identified region, each numbered). (**f**) Overlay of identified regions on the original cropped SRRF image, compared with (**g**)—manually identified regions hand drawn on the raw SRRF image. (**h**) Mask of regions identified by automated processing, where (**i**) shows masks of regions drawn by hand. (**j**) Comparison of region areas calculated from manual and automated analysis, mean ± standard deviation.

In order to quantify the network, a simple method for actin network analysis was tested (Fig. 1a). In FIJI, SRRF images were cropped to an ROI of 10 µm^2^, as centrally in the cell of interest as possible (Fig. 1b). The image was then manually thresholded using Otsu’s method. The threshold was used to generate a binary mask of the network, which was followed by erosion (Fig. 1c,d). A classic watershed segmentation was applied (Fig. 1e), and the resulting regions (‘particles’) (Fig. 1e,f) analysed for a range of descriptors, including area and perimeter. To validate accuracy of the analysis, corrals in the original image were measured manually and compared to the thresholded image. This revealed good correlation in terms of the location of corrals identified (Fig. 1f,g), while providing a more consistent assessment of filament delineation. This results in larger estimates of corral area (Fig. 1h–j). This thresholding approach enables greater degree of consistency between images, and a much higher throughput than via manual assessment. The analysis was also applied to 3D-SIM and TIRF-SIM images of the same regions (Suppl. Fig. S3). This demonstrated that the network analysis tool can detect corrals in all samples, however, as expected, images collected using TIRF imaging performed better due to the good signal to noise ratio and low out of focus light in the images.

**Figure 2:**
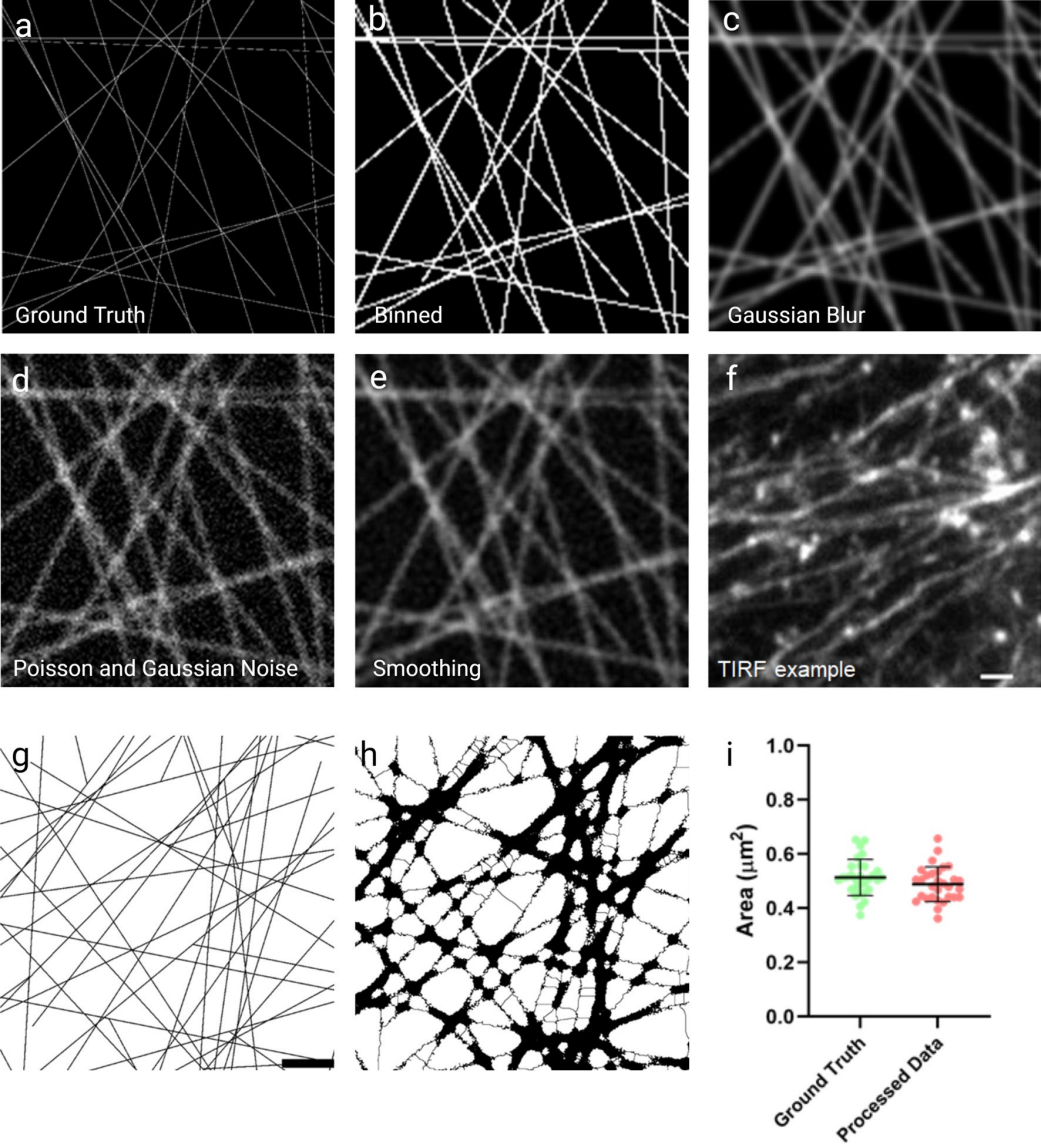
Simulation of meshworks for analysis testing. (**a**) Initial output of 1px wide lines forming a simulated mesh. (**b**) Image (**a**) after dilation of lines to 7px and binning of the image, to match the pixel size of the cameras on our SIM system. (**c**) Convolution with Gaussian blur, as calculated from an estimated PSF based on our optics. (**d**) Application of Poisson and Gaussian noise to mimic noise from the camera. (**e**) Smoothing of (**d**). (**f**) A TIRF image for comparison of the same scale as the simulated data (scale bar = 1 μm). (**g**) Ground truth simulated meshwork (scale bar 1.5 µm), shown in (**h**) after convolution and application of our meshwork analysis. (**i**) Comparison of mean area ± standard deviation of identified corrals from over 30 repeats of network simulation against the ground truth.

In order to validate the reproducibility of the analysis, we applied it to a ground truth data set. Example actin meshworks were simulated and processed to resemble imaging outputs obtained from our system in terms of pixel size and noise. Image simulation was performed in MATLAB 2019b. Filaments were simulated through random generation of start and end points and each individual filament was given a daughter filament, branching at a 70 degree angle (to mimic Arp2/3 nucleated daughter filaments common in cortical actin networks^20^) (Fig. 2a). Within this code, filament number is user definable, and here was kept at 25 filaments per image, with one daughter filament each. Lines were dilated to more closely resemble the 7 nm nature of individual actin filaments and pixels were binned to sizes appropriate for our system and cameras (Fig. 2b) A Gaussian convolution, based on the PSF estimated from our optics, was applied (Fig. 2c). Poisson and Gaussian noise were also applied to give a general approximation of read and shot noise (Fig. 2d), and the image smoothed to give the final simulated image (Fig. 2e). This produced a good representation of our TIRF images of cortical actin networks (Fig. 2f), displaying a similar range of corral sizes within the ground truth data set.

Analysis of these simulated networks using our thresholding analysis showed good correlation in the identification of the same corrals in the ground truth and simulated data (Fig. 2g–i). Analysis of the area of individual corrals gave similar mean ± SD values in the processed data as for the corrals in the ground truth image (ground truth—0.51 μm^2^ ± 0.067, processed data 0.49 μm^2^ ± 0.064) (Fig. 2i). The small, but not statistically significant reduction in area in processed images is likely to be a result of the convolution increasing the relative filament thickness, subsequently reducing the corral area. In a similar vein, most incongruities occur with small corrals that become obscured when the PSF is applied. These small corrals can be considered to be below the resolution limit of the images and can be accounted for by filtering corrals under such a size.

### Cytochalasin D treatment and live cell analysis

**Figure 3:**
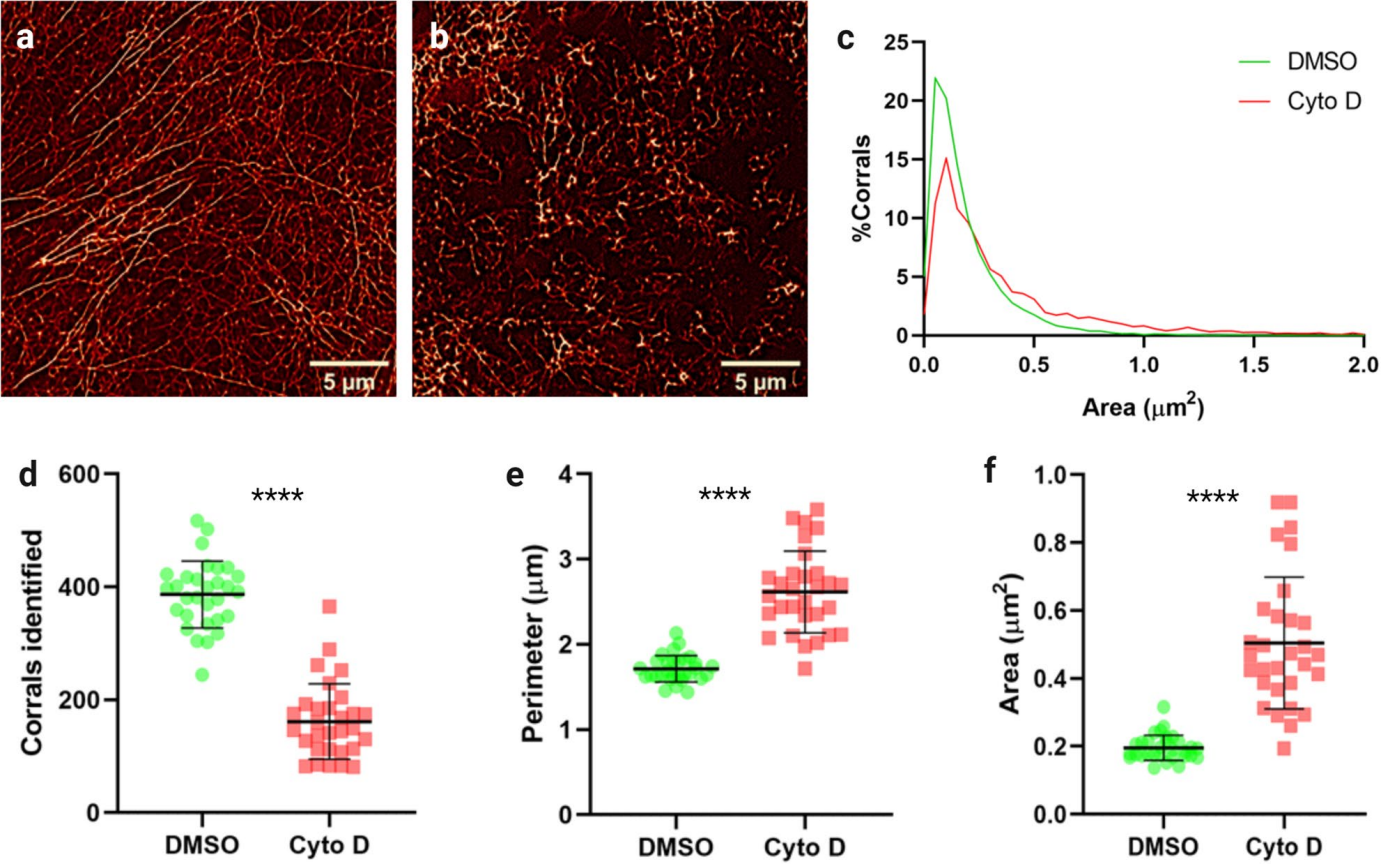
Treatment with cytochalasin D induces significant increase in corral sizes. (**a**) Representative SRRF image of phalloidin labelled actin in DMSO treated cells, and (**b**) cytochalasin D treated cells. (**c**) Histogram of all corral areas for cytochalasin D treated and control cells. (**d**) Number of corrals, (**e**) mean perimeter, and (**f**) mean area of corrals identified in cytochalasin D treated cells versus DMSO control. n = 3, 30 cells per treatment. ****p < 0.0001. All graphs show mean ± standard deviation.

To assess the effect of disruption of actin polymerisation on actin corrals and further validate the analysis technique, cells were treated with 1 µM cytochalasin D—a potent inhibitor of actin filament polymerisation prior to fixation, staining and imaging. Representative images of cells ± cytochalasin D are shown in Fig. 3a,b. Cytochalasin D treatment showed clear disruption of normal cortical actin structure. However, to establish if the meshwork analysis could detect changes to the network, a concentration of cytochalasin D was chosen that did not abolish all actin networks (Fig. 3b). Cytochalasin D treatment significantly increased the size of corrals identified by the analysis workflow (Fig. 3c–f). The mean corral area (control—0.20 μm^2^ ± 0.037, cytochalasin D 0.50 μm^2^ ± 0.19) and perimeter (control—1.71 μm^2^ ± 0.16, cytochalasin D 2.62 μm^2^ ± 0.48) increased significantly (P < 0.0001 for both), while the number of corrals identified in each ROI fell accordingly (control—386.5 ± 59.39, cytochalasin D 161.5 ± 66.91, P < 0.0001).

**Figure 4:**
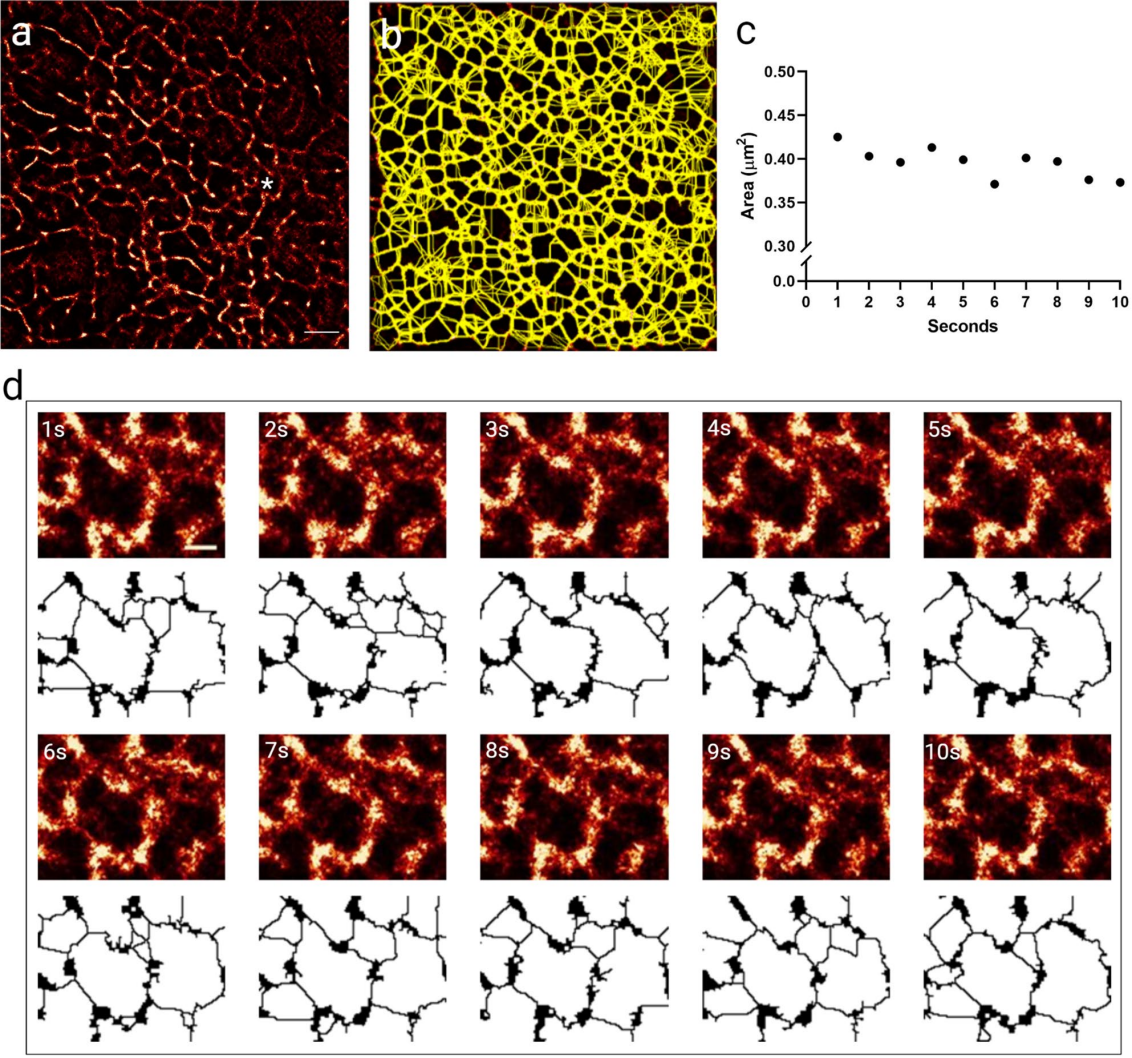
Analysis is applicable to live cell data. (**a**) Example SRRF frame from a 10 s live stack (1 fps) from a LifeAct mEGFP expressing A549 cell. (**b**) Projected overlay of all corrals identified over the 10 frame stack (see Supplementary Video 1). (**c**) Plot of the area of the corral marked with * in a) over time. (**d**) Frame by frame images of the corral marked in (**a**) and plotted in (**c**), showing the raw and processed image.

The analysis is also applicable to live imaging. Using LifeAct mEGFP expressing cells, TIRF images were taken over a 10 s window and SRRF processed, resulting in super-resolved image stacks at 1 fps (Suppl. Video V1). The SRRF movies were analysed frame by frame and specific regions compared over time and to the effect of Cytochalasin D (Fig. 4, Suppl. Fig. S4). Over this short timeframe the actin corrals are fairly stable, however, small fluctuations in their size is observed indicating the dynamic nature of the cortical actin. In agreement with the fixed cell analysis, cytochalasin D treatment reduced the number of corrals present with the mean size of corrals significantly larger than in control cells (Suppl. Fig. S4). Interestingly, there was a trend for increasing corral size in cytochalasin D treated cells over time, even over this relatively short timescale. This demonstrates that the technique, when applied over longer timeframes, could be used to describe cortical actin changes in response to treatment or cell manipulation, for example.

### Expansion microscopy and analysis

While SRRF analysis lends itself to live imaging and is readily adaptable to most imaging setups, resolution is still a limiting factor when considering structures as fine as actin filaments. Given this, we employed expansion microscopy^21^ coupled with SIM imaging to investigate actin networks at resolutions of ~ 40 nm—more comparable to STED’s resolving power, whilst retaining the ability to perform 3D 4-colour labelling for future applications.

**Figure 5:**
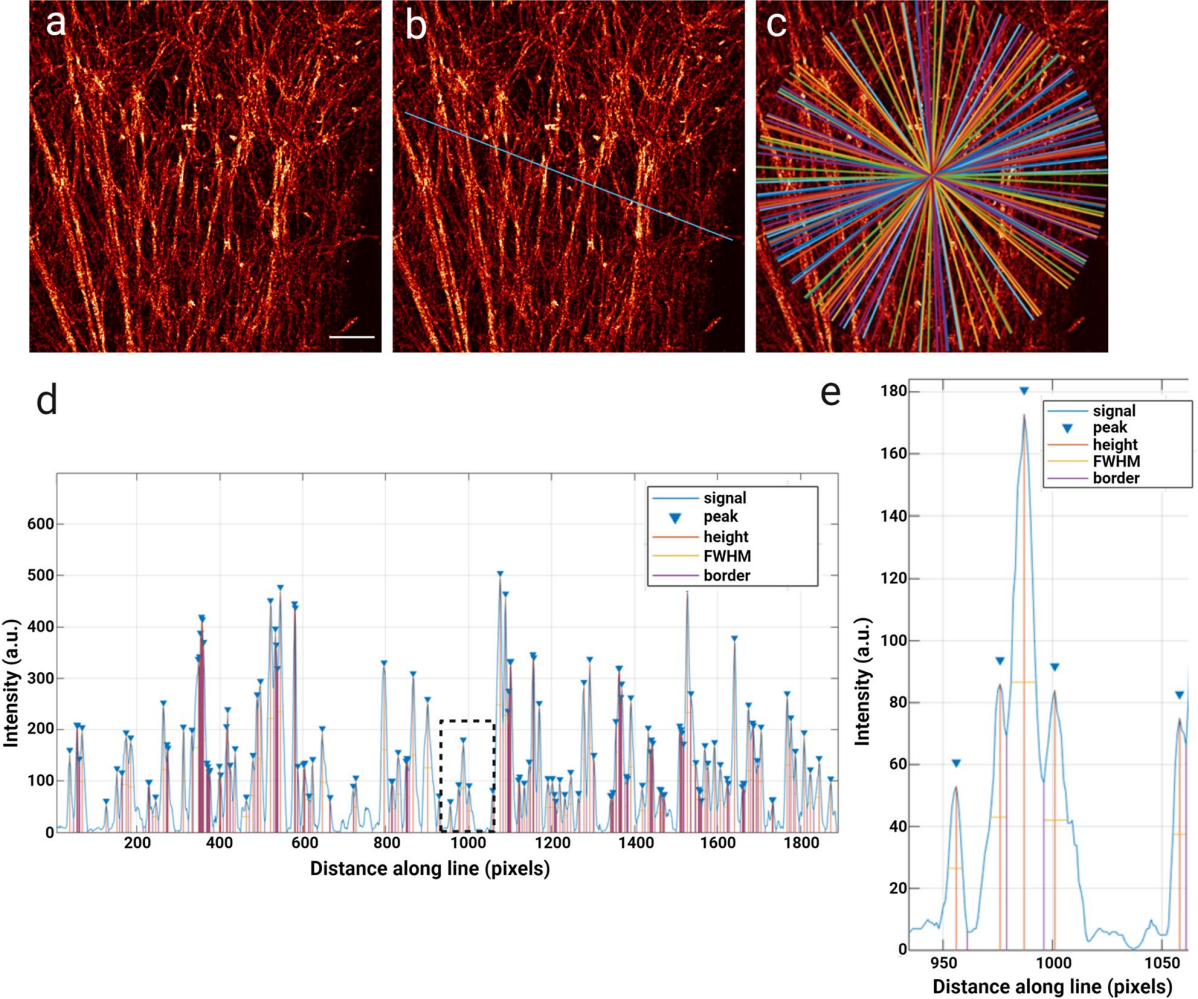
Example of analysis of expanded actin. (**a**) Example of 3D-SIM imaged expanded actin, with a scale bar of 10 μm (uncorrected for expansion factor). (**b**) Randomly generated line across the image for analysis, where intensity along this line is plotted in (**d**). (**c**) Example of the distribution of randomly generated analysed lines when repeated 100 times. (**d**) Normalised intensity line plot of fluorescence intensity (a.u.) vs distance along line for the line shown in (**b**), with peaks identified and height and width denoted as in the key. (**e**) Enlarged region of box in (**d**), showing identification of all distinguishable peaks above a manually set threshold.

In order to visualise actin in expanded cells, the samples were labelled with a phalloidin derivative, Actin ExM. Actin ExM is functionalized with an anchor and therefore compatible with gelation and expansion protocols^22^. Labelling with this reagent in unexpanded cells shows similar actin structures to those labelled by standard fluorescent phalloidins (Supp. Fig. S5). 3D-SIM images of expanded samples clearly show actin structure, with fine meshworks and thicker bundled fibers well labelled (Fig. 5a). 3D reconstructions also highlight the ability of ExM to visualise cortical actin filaments from filaments deeper in the cell (Suppl. Video V2).

Analysis of expanded samples presented a number of challenges. As identified in other studies reduced contrast in ExM can be observed due to several factors (reviewed in^23^). In our experiments some discontinuity in filament labelling was observed, and with the contribution of out of focus light the identification of corrals in ExM images was difficult. Although ExM protocols can be optimised for specific labels and out of focus light can be minimised by adjustment of SIM reconstruction parameters, the meshwork analysis did not work reliably on ExM samples. Therefore, density of filaments in ExM was approximated by examining fluorescence intensity across ROIs of 3D-SIM reconstructions. Randomly oriented lines across full ROIs were drawn (Fig. 5b) and fluorescence intensity graphs generated. These values were normalised and local maxima identified (Fig. 5d), with a threshold to eliminate background, but no threshold on peak prominence, allowing resolution of closely adjacent yet still separate filaments (Fig. 5e). Where peaks were not clearly delineated, a straight line was drawn to separate each peak, and width at half prominence calculated from these (Fig. 5d). Repeating this operation hundreds of times over a single image (Fig. 5c) and comparing the numbers of peaks calculated for each ROI served as a way to compare filament density across the image.

**Figure 6:**
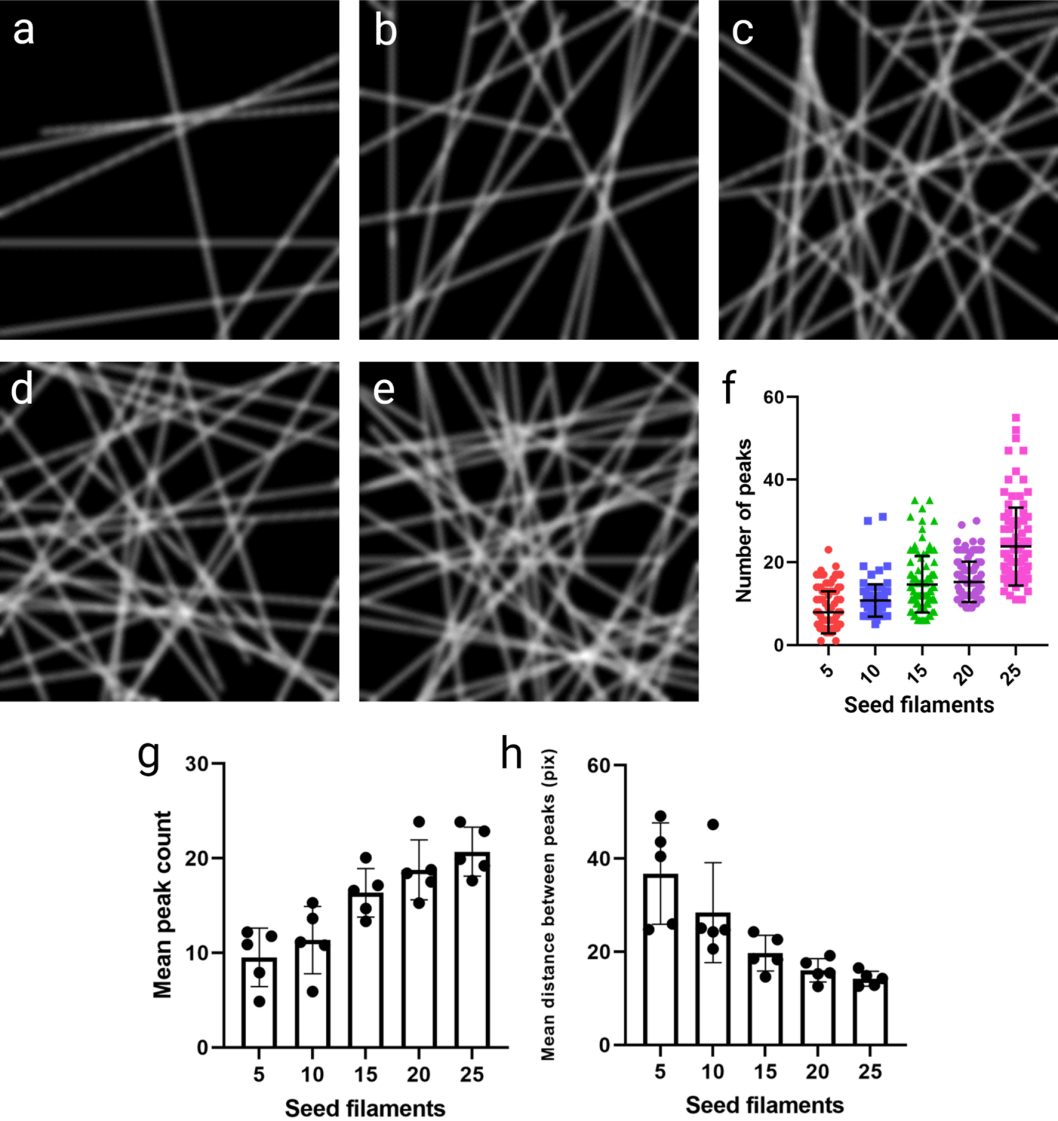
Simulation of meshworks to test density analysis. Simulated meshworks with (**a**) 5, (**b**) 10, (**c**) 15, (**d**) 20, and (**e**) 25 seed filaments, with each filament having the potential to act as a seed to a daughter filament. (**f**) Graph of peaks identified over 100 repeats of density analysis for the images shown in (**a**–**e**). (**g**) Mean peak count and (**h**) mean distance between peaks over 5 individual images for each seed filament condition.

Simulated meshworks were again used to validate performance of the analysis. Using filaments without additional noise, as this would not be representative of 3D-SIM reconstructed images, meshworks of varying densities were generated (Fig. 6a–e) and analysed (Fig. 6f). Application of the analysis showed increase in mean peak numbers as density increased, and a concurrent drop in mean distance between identified peaks (Fig. 6g & h) indicating that the method is robust and able to report on networks of varying density.

**Figure 7:**
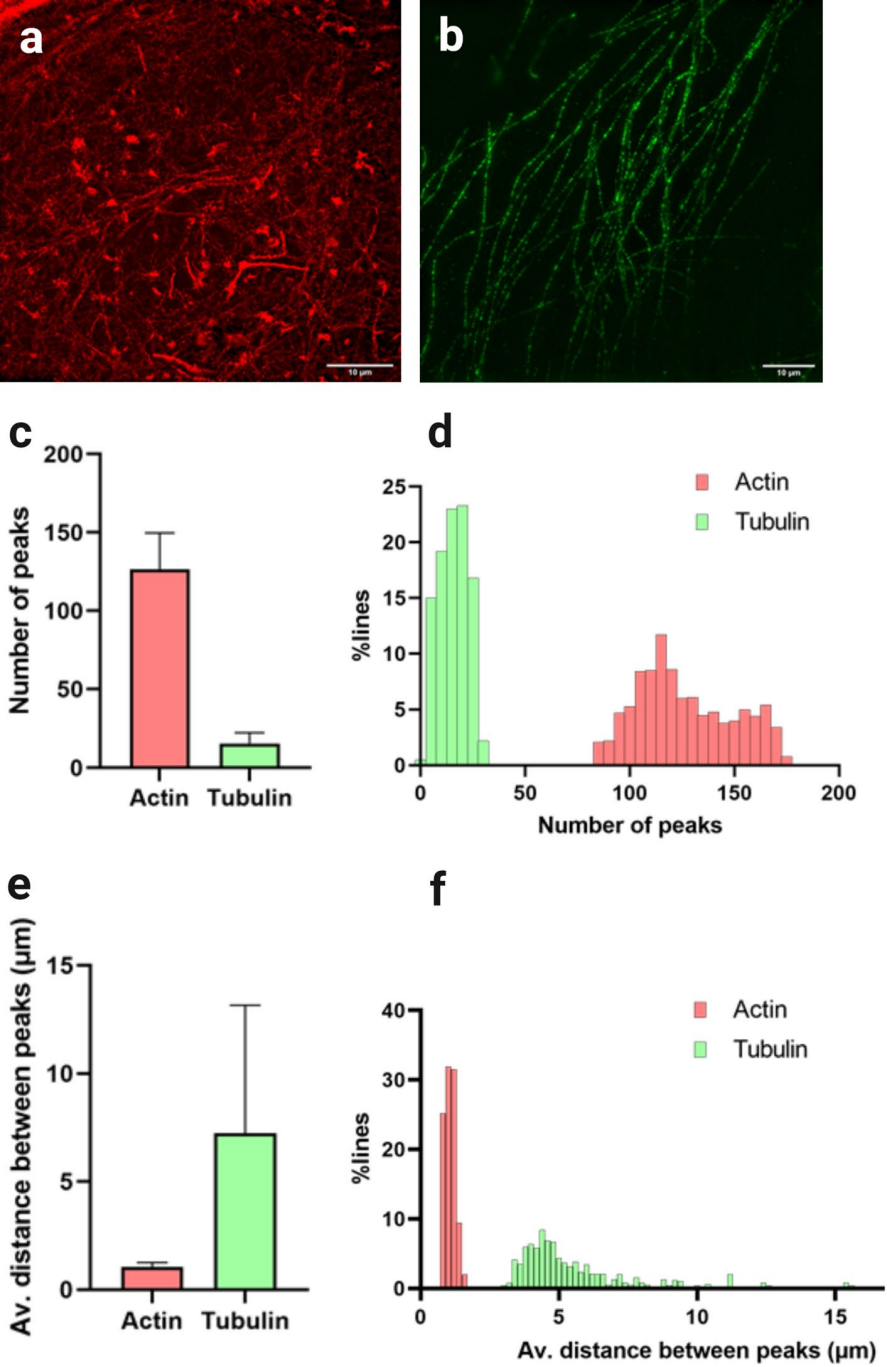
ExM filament analysis distinguishes between small and large meshworks. (**a**) Example 3D SIM image of expanded actin and (**b**) of expanded tubulin (both scale bars 10 μm). (**c**) Mean number of peaks ± standard deviation. Identified over 100 repeats of the analysis for each image, with (**d**) showing a histogram of this dataset. (**e**) Mean distance between identified peaks ± standard deviation over 100 repeats of the analysis for each image, with the histogram shown in (**f**). Lengths in (**e**,**f**) are not corrected for expansion factor.

To demonstrate the power of this analysis, images of A549 cells stained for microtubules and actin and imaged via ExM-SIM (Fig. 7a,b) were subject to filament density analysis. Visually the two networks are seen to comprise different densities of filaments. Quantifying the number of peaks identified showed that the actin network consisted of a higher number of filaments than the microtubule network (Fig. 7c,d). This was confirmed by calculating the distance between peaks (Fig. 7e,f), again showing that the actin networks are more dense than the microtubule ones. This data shows that filament density analysis can determine subtle differences in the organisation of filaments in a cell.

## Discussion

In this study we have developed and tested the application of two workflows for quantifying cortical actin organisation in super-resolved microscopy images. It contrasts other actin filament analysis methods in that, rather than skeletonising the filaments and calculating filament length, filament branching, branching angle, our SRRF analysis assesses the shapes and sizes of the meshwork.

Accurate quantitation of the actin meshwork depends on the generation of reliable, quality super-resolved images. SRRF imaging performs well on actin, giving clear and reproducible structures that correlate well with the TIRF raw data and as quantified by SQUIRREL analysis. However, false sharpening and the collapsing of adjacent objects is a common issue in SR reconstructions, including in common single molecule localisation microscopy reconstruction algorithms^24^. Care should be taken when reconstructing super-resolution images and appropriate quality control checking performed. The application of SQUIRREL^19^ here helped to identify regions of possible error in reconstructions. These were mostly observed in brighter, sharper regions of the reconstruction. However, it is important to note that the error maps are only internally relative; RSP values give a more inter-experiment and inter-technique comparable value, and in our experiments RSP values were consistently high.

The meshwork analysis developed here performs well on SRRF data, and on data simulated to represent the binning and pixel size of SRRF data. The comparison of simulated versus ground truth data also highlighted that the analysis can underestimate some areas by over segmenting as well as missing smaller regions that sit below the resolution limit. This is, however, an issue inherent to any resolution limited image, and by filtering out very small corrals (i.e. those below the resolution limit) from further analysis. With this caveat in mind, the analysis performed well, identifying similar regions, which were also similar in size, between our simulated and ground truth data. To further test the analysis, and to see if it could detect subtle changes in the actin network, we treated cells with the actin disruptor cytochalasin D. From our analysis, the effect of cytochalasin D on the samples was as predicted—a visible disruption of normal cortical actin structure, with the persistence of some, especially thicker, filaments. Cytochalasin D acts by binding the barbed end of actin filaments, inhibiting polymerisation and dissociation from this end. Typically, cytochalasin D induces the formation of denser focal accumulations of actin, likely by interrupting normal anchoring of filaments by capping proteins^25^. As such, normal membrane associations should be severely disrupted, as indicated by our data. The accurate representation of cytochalasin D effects by our analysis workflow therefore strengthens justification for broader application and to assess subtle changes in cortical actin network organisation in response to inhibitors or stimulation.

Skeletonisation analysis methods work well for continuous, well separated filaments, such as microtubules, intermediate filaments, or even thicker actin stress fibers (see Zhang et al.^26^ for implementation of their technique SFEX to quantify actin stress fibers in TIRF images). When considering super resolved fine actin meshworks, however, discontinuity and reduced fluorescence intensity is an inherent issue. This can lead to artefacts like mismatching of filament segments and artificial removal of sparser filaments. By focussing on the gaps the actin leaves rather than trying to extract information from the filaments themselves, artefacts introduced in the thresholding steps can be somewhat mitigated. A recent preprint describes an algorithm called FiNTA that performs more advanced and accurate filament tracing than typical skeletonisation^27^, but this technique is reported to perform best on filaments of uniform thickness.

The simple technique we describe should be easily applicable to other super resolution techniques, such as SIM and STED, but better options are available for SMLM. As a rule of thumb, where analysis can be applied directly to the point cloud data generated in single molecule techniques rather than images reconstructed from this data, it should be. This retains the maximum information and resolution gained by using these techniques. Peters et al.^12^ developed an algorithm to perform filament tracing from the spatial point patterns generated in SMLM imaging of actin, allowing extraction of information directly from the point cloud rather than the reconstructed image. Identification and differentiation of fibrous and clustered structures in SMLM data is also possible using Williamson et al.’s^14^ machine learning based analysis approach.

Expansion microscopy offers a far superior resolution to SRRF, albeit only in fixed cell contexts. Underutilised in the study of actin, expansion microscopy is shown here to faithfully preserve both bundles and fine actin structure, with resolution almost comparable to SMLM when used in conjunction with SIM^28^. Where expansion could be arguably an improvement on single molecule techniques is the potential for simple and rapid multiplexing of labels. The nature of ExM as, essentially, a modified standard immunofluorescence technique, means that imaging four spectrally distinct labels in one sample is relatively easy to achieve. While techniques like DNA-PAINT^29^ are more easily adaptable than dSTORM or PALM for multi-colour imaging, this comes at a significant time cost—a single image can take hours to acquire. In addition, as expansion microscopy sample preparation optically clears the sample, thereby making samples more amenable to super-resolution 3D imaging, expansion is a strong candidate for investigating complex actin structures in 3D in cells. Recently, Park et al.^30^ used anti-fluorophore antibodies to image actin labelled with standard Alexa-488 conjugated phalloidin, which, while a different approach to the one taken here, indicates that techniques for actin imaging in expanded samples are growing. Whilst filament tracing routines have been applied to microtubules post expansion^27^, the relative density and continuity of fine actin filaments continues to restrict application. The method we describe here uses fluorescence intensity fluctuations as a proxy for filament density and allows these complex filament networks to be quickly quantified. Importantly, while this method allows relative comparison between images, it is not a way to calculate total filament number in a field of view. Samples will contain filaments that start or end within the image, meaning not every filament will be captured in each repeat of the analysis. As the results are averages, this will lead to consistent underestimation of true filament number (Suppl. Fig. S6). However, it provides a quick, robust and useful metric when comparing cortical actin network densities, and can be used to complement or extend, more complex filament tracing analyses.

In conclusion, we demonstrate two simple analysis methods for quantifying cortical actin networks in super-resolved microscopy images. These methods allow for quick, reproducible quantitation of actin corral number and size in SRRF or SIM images, and for quantitation of filament density in ExM images. These methods are amenable to batch processing of large data sets, and to adaptation for live cell analysis of actin dynamics.

## Methods

### Cell preparation for SRRF and SIM imaging

A549 cells were plated in #1.5 high tolerance glass bottomed Ibidi dishes and allowed to attach for minimum 18 h. Where transfection was required for live imaging, cells were plated and transfected the next day with PEI and LifeAct mEGFP plasmid (Supplementary Fig. S7) and allowed to express for 48 h prior to imaging. For actin disruption, 1 µM Cytochalasin D was applied for 30 min at 37 °C, with equivalent volumes of DMSO as vehicle control, to a final concentration of no more than 0.05%. Cells were fixed for 15 min in 4% PFA in PEM buffer (0.8 M PIPES (pH 6.95), 4 mM EGTA, 1 mM MgSO_4_) that had been pre-warmed to 37 °C, and washed 3 × briefly with PBS. Samples were quenched with 50 mM NHCl_4_ for 10 min, followed by permeabilization with 0.1% Triton X-100 for 5 min and 3 × brief PBS washes. Cells were then labelled with 6.6 nM Alexa Fluor 488 Phalloidin (Invitrogen) for one hour, with 3 × brief PBS washes prior to imaging. Imaging was performed in PBS.

Samples were imaged using the Nikon N-SIM-S system (Ti-2 stand, Cairn TwinCam with 2 × Hamamatsu Flash 4 sCMOS cameras, Perfect Focus, Nikon laser bed 488, 561 and 647 nm excitation lasers), Nikon 100 × 1.49 NA TIRF Apo oil objective or Nikon 100 × 1.35 NA Silicone oil objective, for expansion gels). SIM data was reconstructed using NIS-Elements (v. 5.21.03) slice reconstruction. Representative reconstructed SIM data was assessed by way of the FIJI plugin SIMCheck^31^. SRRF images were reconstructed from 100 frame bursts of TIRF images, taken at optimal exposure for each sample, as structure rather than intensity was being assessed. For each repeat, a minimum of 10 cells were imaged. Live samples were imaged in TIRF at 100 fps over 10 s, resulting in a SRRF movie at a rate of 1 fps.

### Expansion microscopy

Protein retention expansion microscopy was performed according to the technique published by Tillberg et al.^21^. Cells were seeded as above, and fixed with 4% PFA + 0.1% glutaraldehyde in PEM, prewarmed to 37 °C. Fixation was followed by 3 × PBS washes, quenching of free aldehydes with 50 mM NHCl_4_ for 10 min, 3 × PBS washes, and a 5 min extraction with 0.1% Triton X-100. Where immunofluorescence labelling was necessary, samples were then blocked with a 0.1% Bovine Serum Albumin (BSA)/2% goat serum blocking buffer for 1 h before incubation with the primary antibody, mouse monoclonal anti-α-Tubulin (Sigma-Aldrich), diluted to 1 µg mL^−1^ in blocking buffer, again for 1 h. After 3 × PBS washes, incubation with the secondary antibody (goat anti-mouse Alexa Fluor 488, A11001, Invitrogen) proceeded for a further hour.

After 3 × PBS washes, all slips were then anchored with 0.1 mg mL^−1^ Acryloyl-X, SE (6-((acryloyl)amino)hexanoic acid, succinimidyl ester (abbreviated as AcX) at room temperature overnight. Slips were washed once in PBS and labelled with Actin ExM (Chrometra) at 1 unit/coverslip for 1 h. Anchoring with AcX prior to Actin ExM labelling was essential to ensure undistorted retention of the Actin ExM probe post expansion. Gelation proceeded immediately after 3 brief PBS washes.

To make the gel, monomer solution (1 × PBS, 2 M NaCl, 8.625% (w/w) sodium acrylate (SA), 2.5% (w/w) acrylamide (AA), 0.15% (w/w) *N*,*N*′-methylenebisacrylamide (BIS)) was prepared and aliquoted, storing at − 20 °C until use. Concentrated stocks (10% w/w) of tetramethylethylenediamine (TEMED) accelerator and ammonium persulfate (APS) initiator were added to the monomer solution up to 0.2% (w/w) each. APS was added last and all solutions were kept on ice to prevent premature gelation. After thorough vortexing of the gel mixture, droplets were pipetted onto parafilm covered glass slides placed in a humid chamber. Coverslips were quickly inverted onto the droplets and gelation was allowed to proceed at room temperature for approximately one minute, before moving to incubate at 37 °C in the dark for 2 h. It is essential that the humid chamber remains moist during gelation in order to reduce the risk of the gel ripping from the coverslip prematurely in later manipulations. Once gelation was complete, gelled coverslips were carefully removed from the parafilm with flat tweezers and placed in a 6 well plate. Digestion buffer (50 mM Tris (pH 8), 1 mM EDTA, 0.5% Triton X-100, 0.8 M guanidine HCl) was supplemented with Proteinase K (New England Biolabs), diluted to 8 units mL^−1^. Gelled coverslips were submerged in this digestion buffer overnight at room temperature. Following digestion, gels were transferred to excess deionised water and incubated. Water was replaced until expansion plateaued at an approximate diameter of 5.4 cm for the full gel.

For SIM and widefield imaging, high precision glass bottomed Ibidi dishes were fully coated with poly-l-lysine (Sigma-Aldrich) and dried on a hotplate at 95 °C. Once fully expanded, a portion of gel was cut with a rectangular coverglass to fit the dish. Excess water was wicked from the gel with tissue to ensure adherence to the coverslip and reduce movement of the gel. The gel was gently pressed once placed on the coverslip to remove bubbles and facilitate firm attachment. A drop of water was added to the top of the mounted gel to minimise shrinkage. Samples were imaged on the Nikon N-SIM-S system as described above.

### Image analysis

Image analysis was performed using Fiji^32^ and Matlab (2019b, v 4.0). Custom written Matlab scripts used for meshwork simulation and line plot analysis are available on github (https://github.com/biologevie/actin-analysis). A workflow for Fiji analysis is shown in the results section below. SRRF data was assessed with NanoJ-SQUIRREL^19^.

### Statistics

All graphs were made and statistical analyses performed using GraphPad Prism 8. Differences between paired groups were assessed using Students T-Test.

## Supplementary Information

Supplementary Information 1.

Supplementary Video 1.

Supplementary Video 2.

## Data Availability

The datasets generated during and/or analysed during the current study are available from the corresponding author on reasonable request.
