# Supplementary material for: Simple methods for quantifying super-resolved cortical actin
Source: Sci Rep. 2022 Feb 17;12:2715. doi: 10.1038/s41598-022-06702-w (PMC8854627; doi:10.1038/s41598-022-06702-w)
Supplement: Supplementary file 1 — Supplementary Information 1. [file 41598_2022_6702_MOESM1_ESM.docx]

**Simple methods for quantifying super-resolved cortical actin**

**^1,2^Evelyn Garlick, ^1,2^Emma L. Faulkner, ^2,3^Stephen J. Briddon ^1,2^Steven G. Thomas***

^1^ Institute of Cardiovascular Sciences, College of Medical and Dental Sciences, University of Birmingham, Edgbaston, Birmingham, UK, B15 2TT

^2^ Centre of Membrane and Protein and Receptors (COMPARE), University of Birmingham and University of Nottingham, Midlands.

^3^ Division of Physiology, Pharmacology and Neuroscience, School of Life Sciences, University of Nottingham, Nottingham, UK.

* Author for correspondence; s.thomas@bham.ac.uk

**Supplementary Information included in this document**

Supplementary figures S1-S7

Supplementary video V1 & V2 legends

**
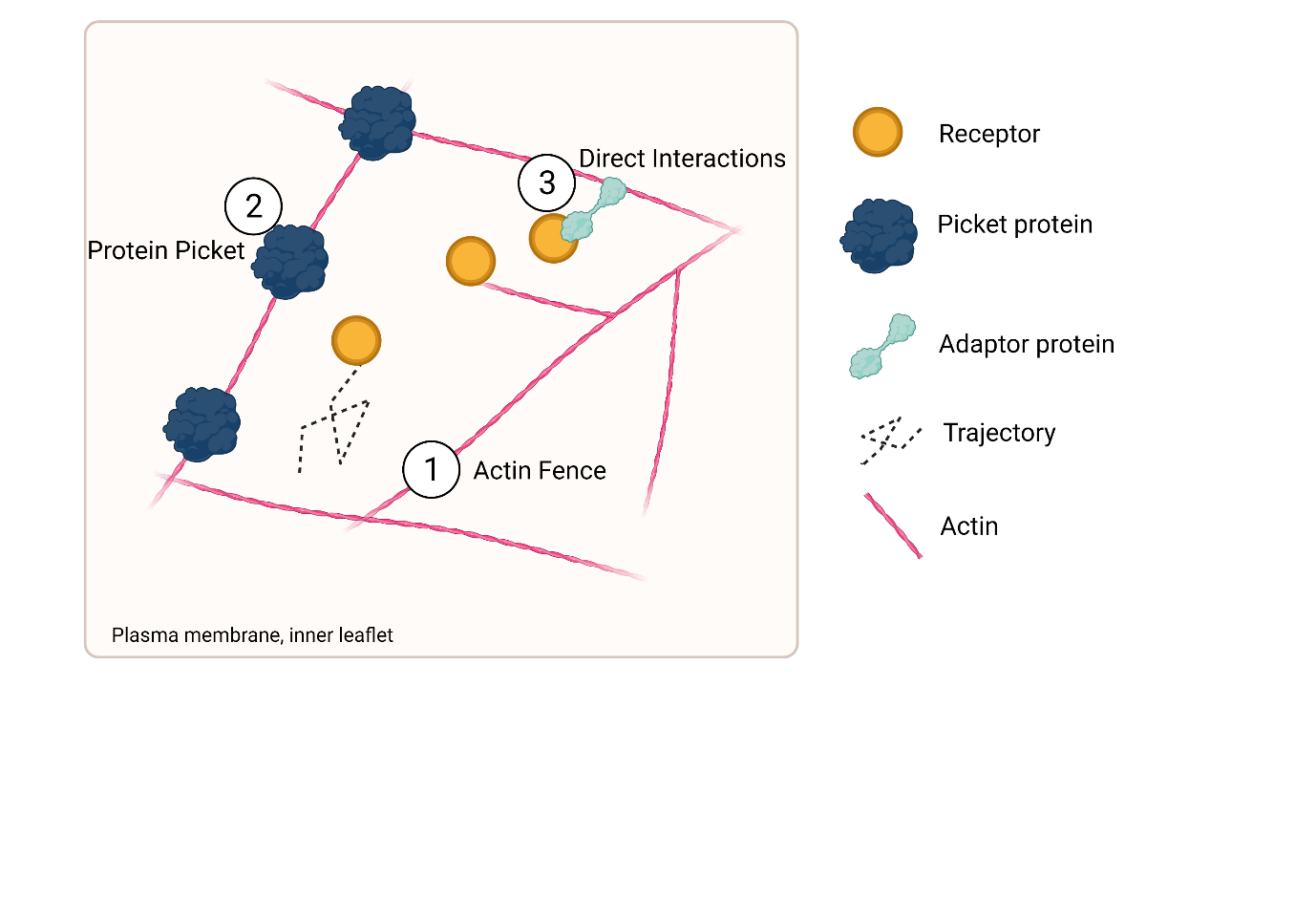
**

**Supplementary Figure S1 - Picket fence model of membrane organisation**. Diagram showing proposed nature of the picket fence model on the inner leaflet of the plasma membrane. Potential methods of receptor confinement include 1) direct physical impediment of receptor movement by the actin filaments, 2) alteration to lipid packing around picketing proteins, and 3) direct interaction with actin filaments, with or without adaptor proteins.


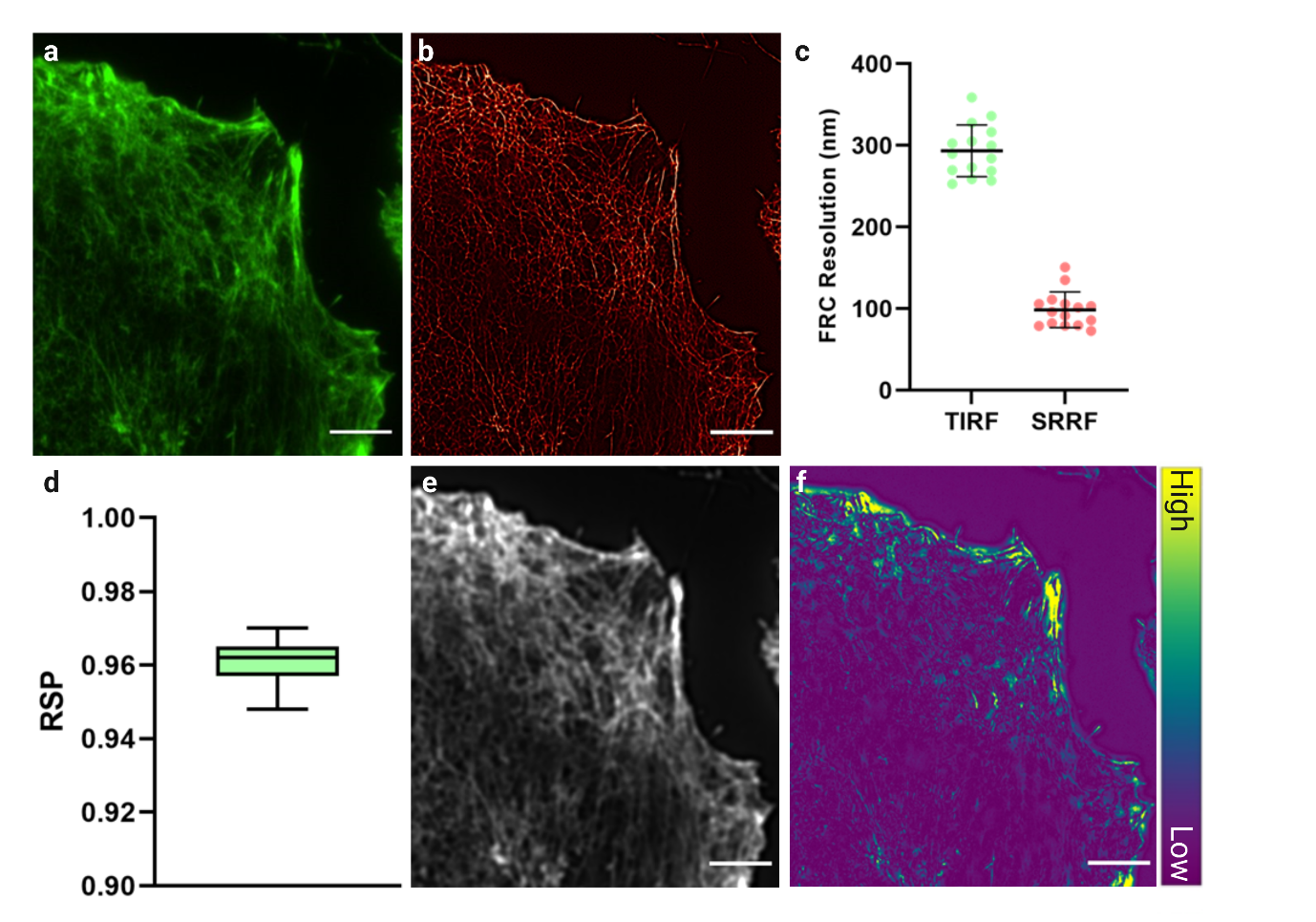


**Supplementary Figure S2 - SRRF reconstructions show minimal error compared to TIRF images.** a) Representative TIRF image of actin in A549 cells labelled with phalloidin Alexa 488. b) SRRF reconstruction from 100 frames of TIRF data represented in a). c) Mean Fourier ring correlation (FRC) resolution ± standard deviation for the TIRF and SRRF images (n = 15 images from 3 independent experiments). d) Plot showing mean resolution scaled Pearson’s correlation coefficient ± standard deviation for 15 images over 3 independent experiments, as calculated with NanoJ-SQUIRREL. 1 is total correlation and -1 is total anticorrelation. e) Convolved image from b) generated by Nano-J SQUIRREL to calculate error. f) Error map, assessing e) vs a). Indicating artefacts in reconstruction occur in areas of thicker and more dense filaments.


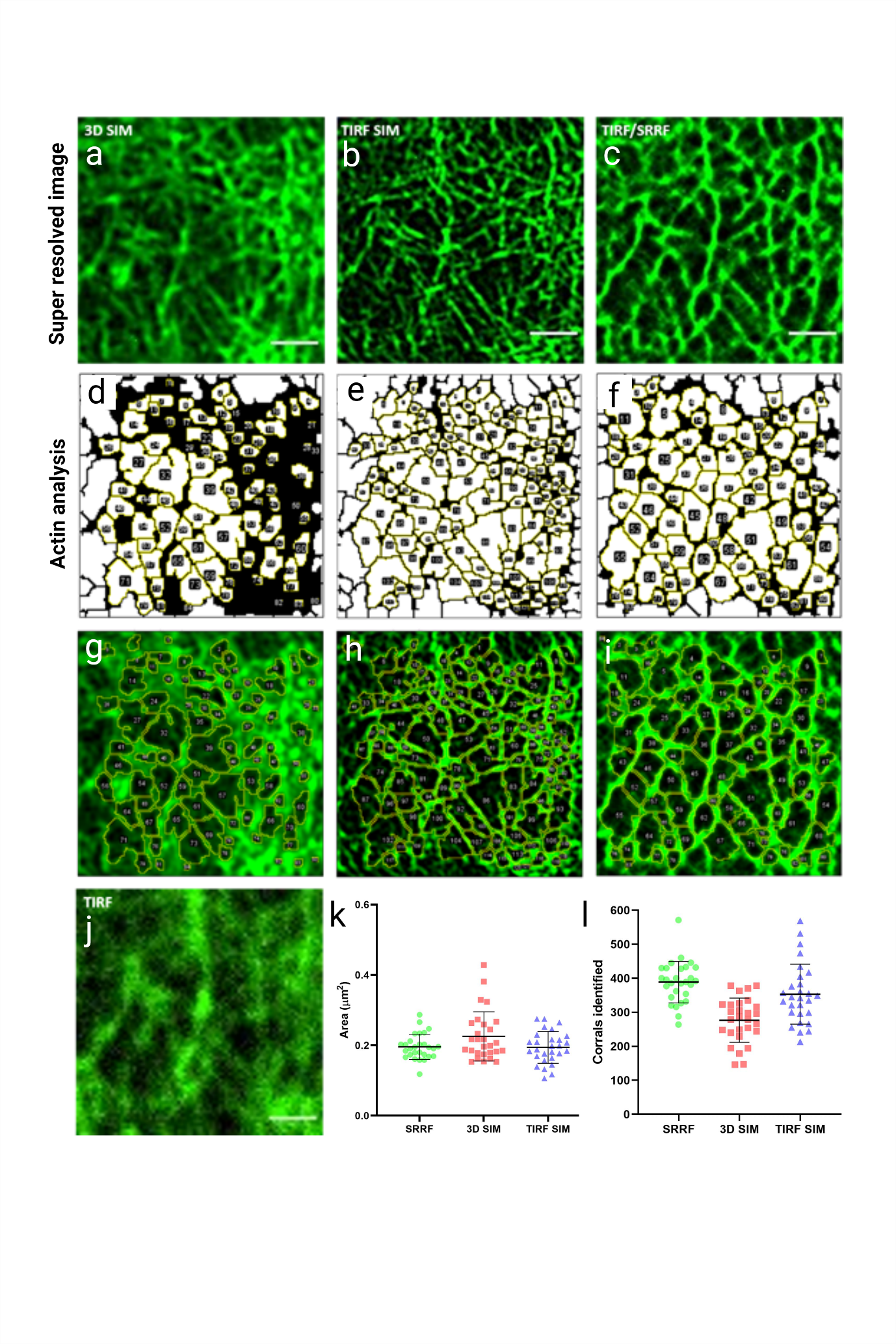


**Supplementary figure S3 -** **Analysis is applicable to other super-resolution techniques with good SNR**. a) 3D SIM, b) TIRF SIM, and c) SRRF super resolved images of phalloidin-488 labelled actin, with accompanying corral analysis (d-f) overlaid on the ROI (g-i). j) Raw TIRF image corresponding to the ROI imaged in a-c. Mean area (k) and number of corrals identified (l) by automatic analysis of 30 ROIs per technique, indicating that 3D-SIM underestimates corrals while both TIRF based techniques perform robustly.


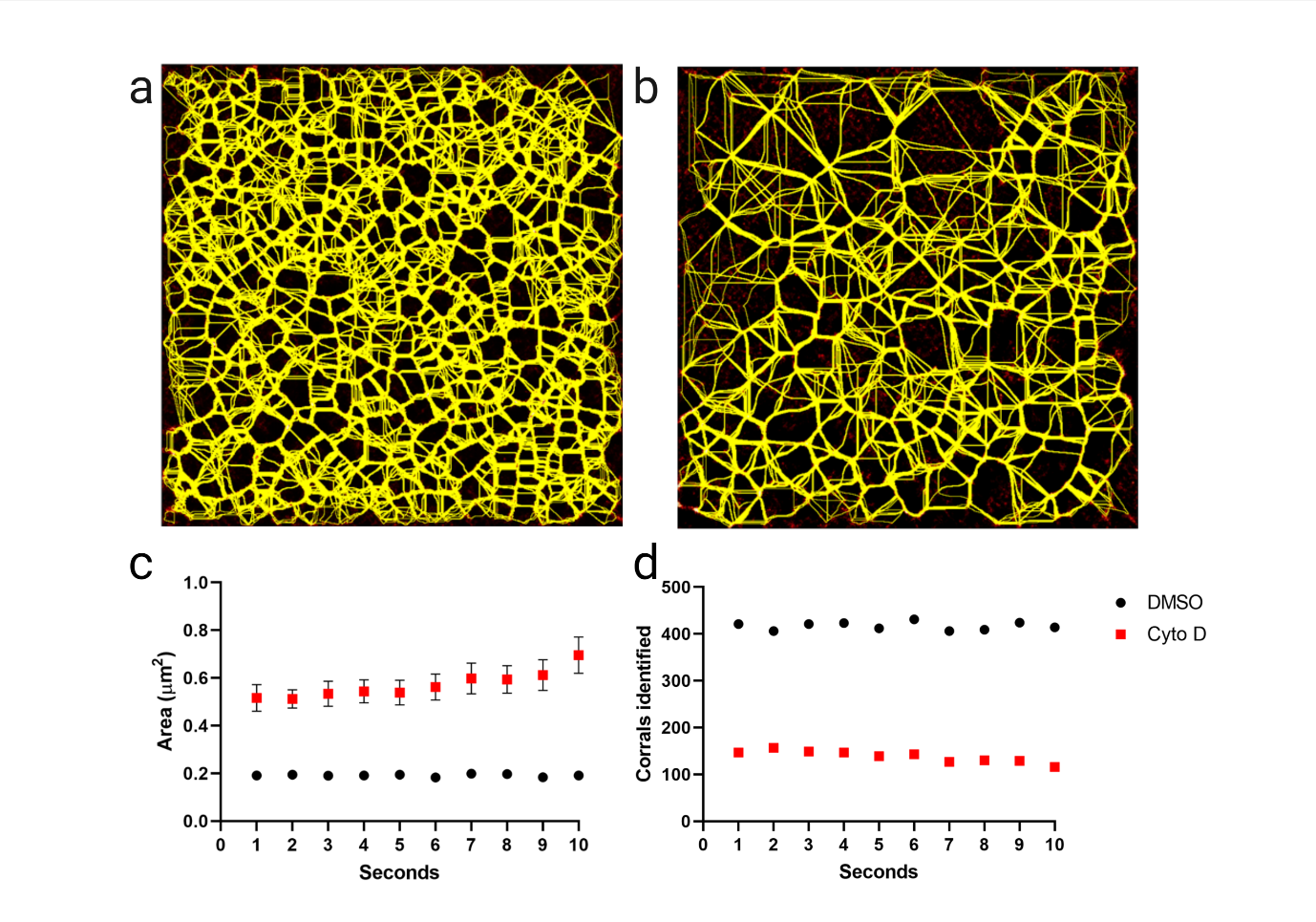


**Supplementary figure 4 - Analysis of live cell data.** Projections of all corrals identified in a 10 frame movie for DMSO treated (a) and Cyto D treated (b) cells. c) Mean area (± SEM) and d) corral count are plotted over time.


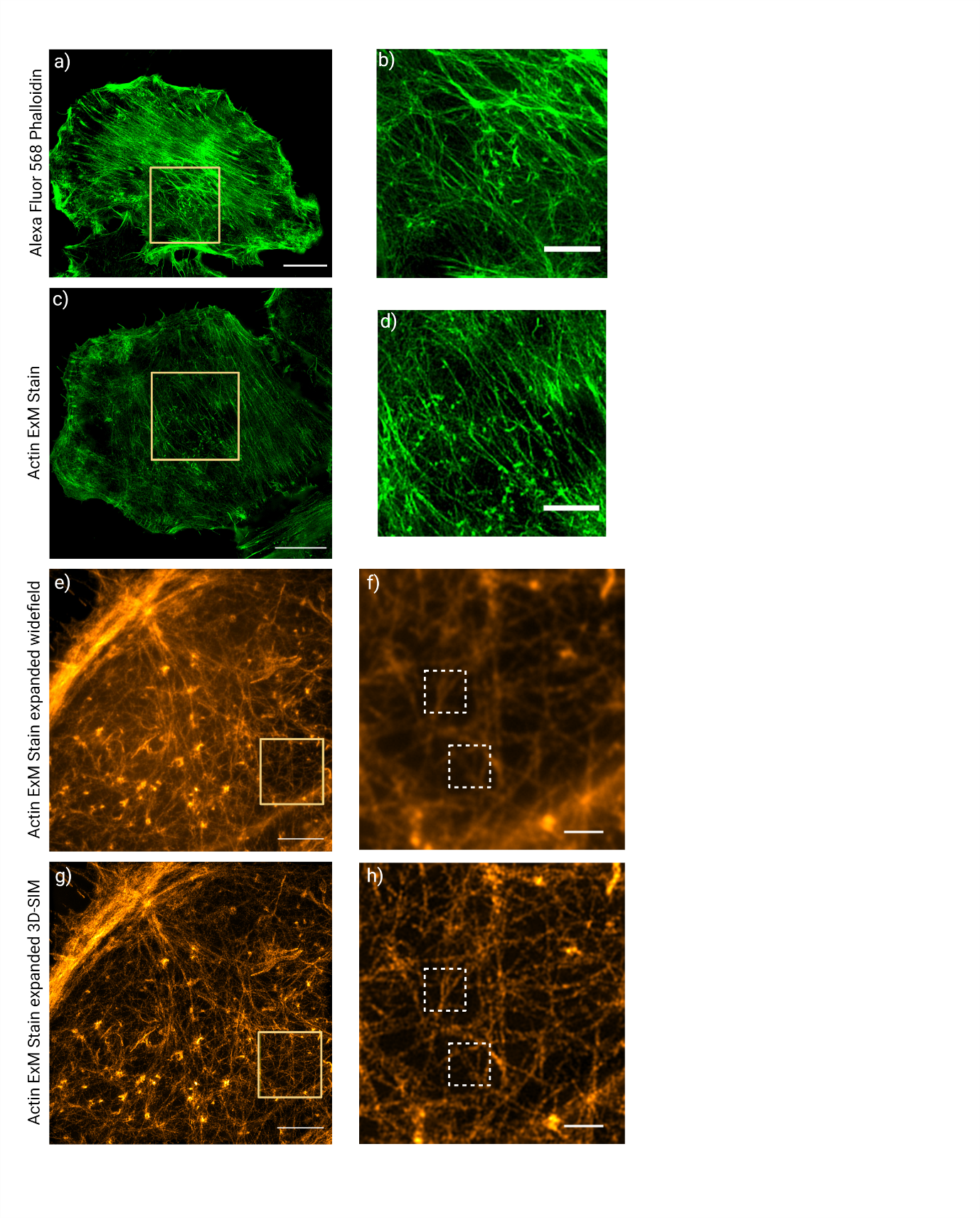


**Supplementary Figure S5 - Comparison of actin labelling and imaging modality for ExM samples**. a) Representative 3D SIM reconstruction of an A549 cell labelled with standard AF 568 phalloidin, with b) showing a zoom of the boxed region of the cell. c) Representative 3D SIM reconstruction of an unexpanded, un-gelled A549 cell labelled with Actin ExM, with d) showing a zoom of the boxed region of the celll. e) Representative widefield image of an Actin ExM labelled and expanded cell. f) Enlargement of the yellow boxed region in e). g) 3D SIM reconstruction of the same Actin ExM labelled expanded cell in e), with h) showing an enlargement of the yellow box. Boxed regions in f) and g) highlight regions of visibly improved resolution in the 3D SIM image. (Scale bars: a, c, e & g = 10 μm, b & d = 4 μm, f & h = 2 μm. Scale bars are not adjusted for expansion factor for images e-h.)


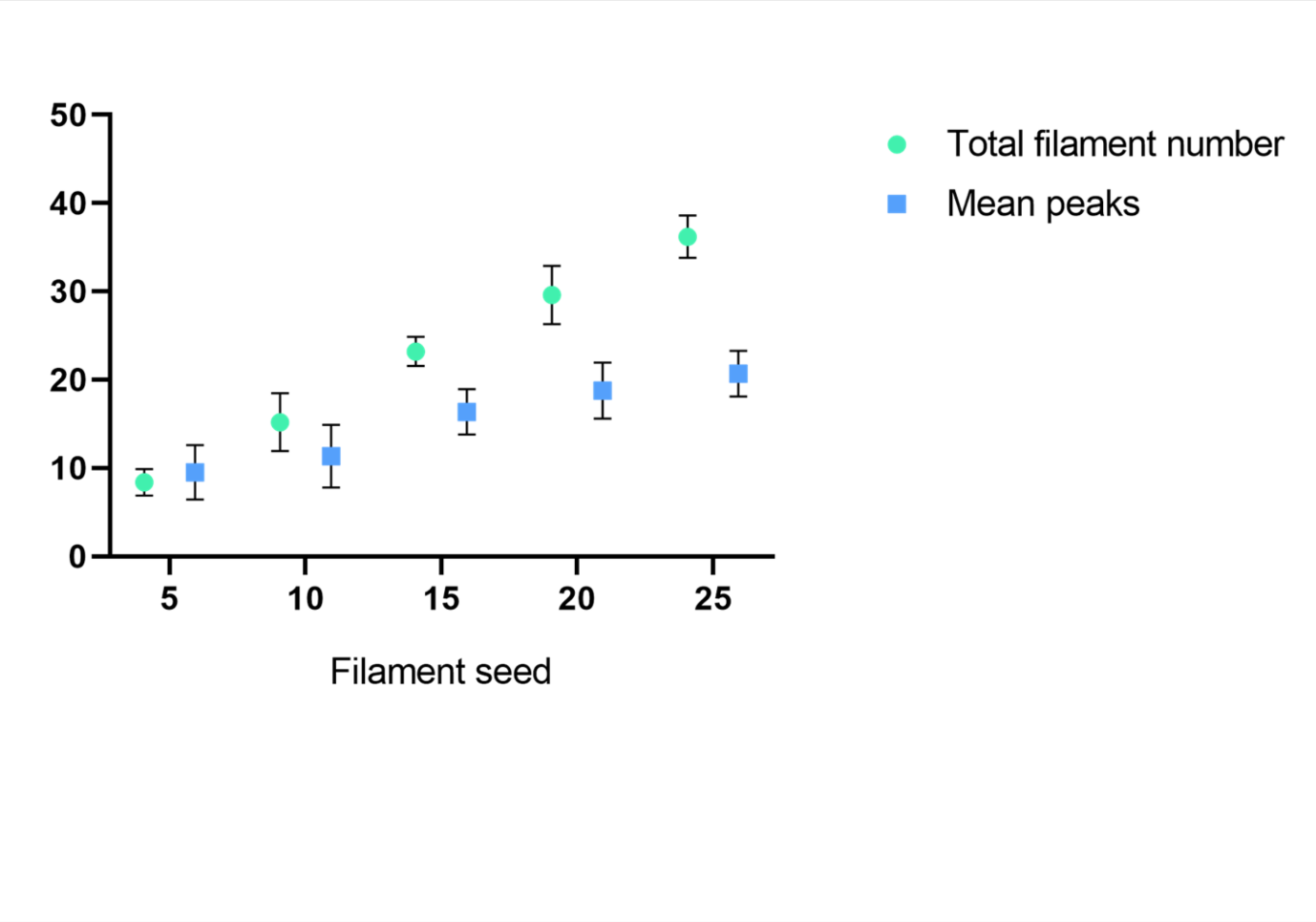


**Supplementary figure 6 - Density analysis underestimates total filament number.** The graph shows the total number of filaments identified per ROI for 5 individual simulated images for each seed density, compared with the mean number of peaks identified in the same images using our density analysis.


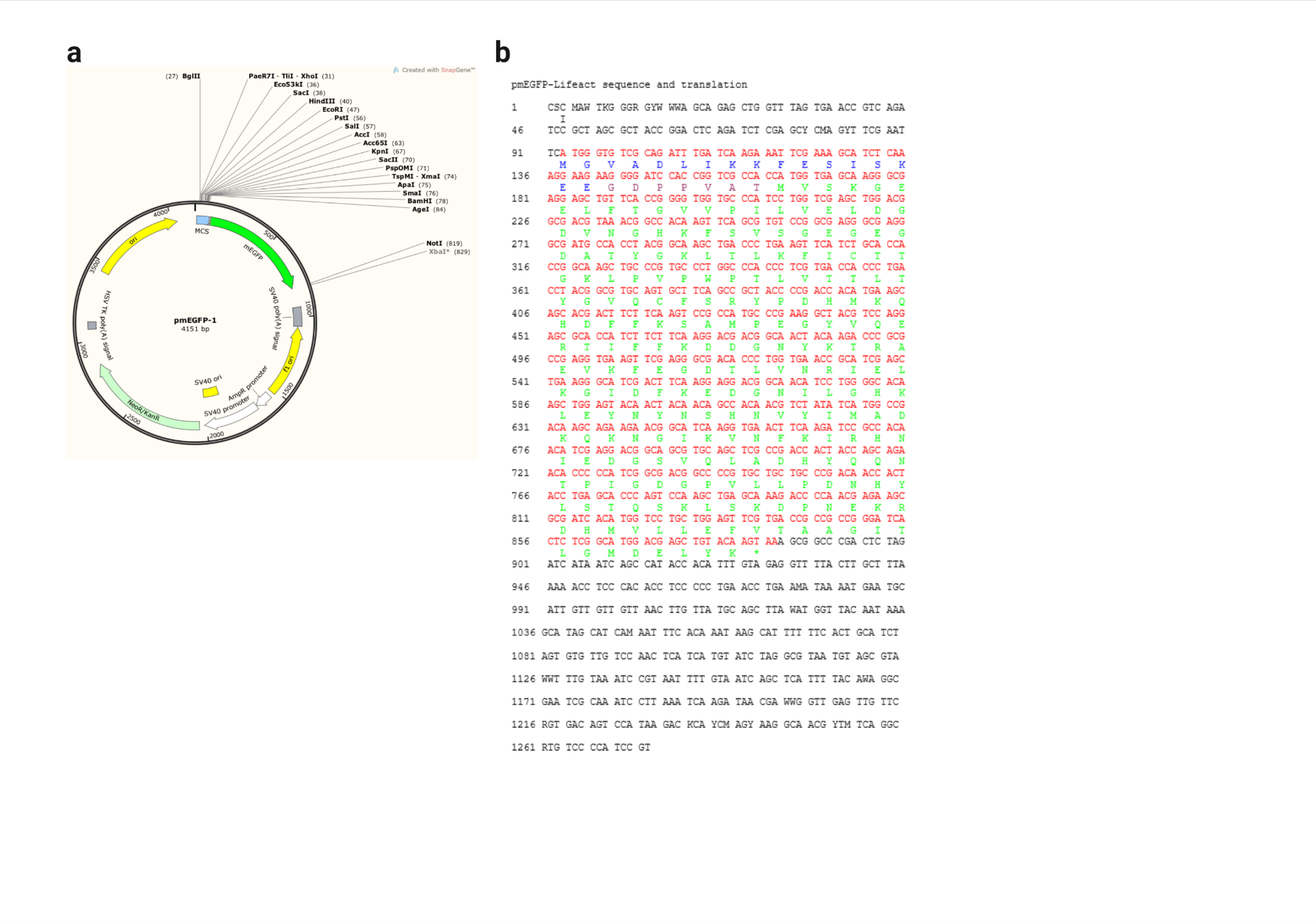


**Supplementary figure 7 – Lifeact-GFP plasmid details.** a) Plasmid backbone map for the pmEGFP-N1 plasmid used to transiently express Lifeact-GFP. b) Sequence of the portion of the plasmid containing the Lifeact-GFP showing the 17 amino acid Lifeact peptide (blue), the 6 amino acid linker (purple), and GFP (green).

**Supplementary video V1**

Movie showing processed SRRF time lapse images of actin network (left hand side) and the outputted actin corral analysis (right hand side). The SRRF movie represents 10 seconds of imaging at 10 ms exposure time. SRRF analysis was performed on 100 frame blocks giving a final 10 frame movie at 1fps SRRF.

**Supplementary video V2**

Movie showing a portion of an Expanded A549 cell labelled with Actin ExM and imaged using 3D-SIM. Depth colour scale applied allowing actin filaments at different depths to be identified.
